# Exploring the Immunopathogenesis of Pregnancy With COVID-19 at the Vaccination Era

**DOI:** 10.3389/fimmu.2021.683440

**Published:** 2021-07-08

**Authors:** Dan Lv, Jing Peng, Rui Long, Xingguang Lin, Renjie Wang, Di Wu, Mengzhou He, Shujie Liao, Yun Zhao, Dongrui Deng

**Affiliations:** ^1^ Department of Obstetrics and Gynecology, Tongji Hospital, Tongji Medical College, Huazhong University of Science and Technology, Wuhan, China; ^2^ Department of Obstetrics, Maternal and Child Health Hospital of Hubei Province, Tongji Medical College, Huazhong University of Science and Technology, Wuhan, China

**Keywords:** immunopathogenesis, SARS-CoV-2, COVID-19, pregnant, vaccine

## Abstract

Since December 2019, Wuhan, China, has experienced an outbreak of coronavirus disease (COVID-19), which is caused by the severe acute respiratory syndrome coronavirus 2 (SARS-CoV-2). Pregnant women are deductively considered to be in immunosuppressive condition for the safety of semi-allograft fetuses, which increases the risk of being infected by the virus. In this review, we analyzed the unique immunological characteristics of pregnant women and reviewed their known outcomes at different trimesters from the perspective of underlying mechanisms that have been studied and speculated so far.

## Introduction

Since December 2019, the world has experienced a new disease termed COVID-19 (1), which is caused by SARS-CoV-2 according to its taxonomy proposed by The International Committee on Taxonomy of Viruses (2). Until March 23, 2021, over 122 million cases worldwide have been confirmed; the death toll has reached over 2.7 million (3). SARS-CoV-2 is the third coronavirus that has led to a fulminant outbreak, the others being SARS-CoV and MERS-CoV. The diameter of SARS-CoV-2 ranges from 60 to 140 nm. The genomic sequence of SARS-CoV-2 is 96.2% homologous with *Rhinolophus affinis* termed RaTG13 (4), which originated from bats. The high similarity between SARS-CoV-2 and RaTG13, together with their close cluster in phylogenetic analysis makes bats a deduced host of SARS-CoV-2. The role of intermediate host in the outbreak of a pandemic also matters, as palm civets do in SARS-CoV and dromedary camels in MERS-CoV. Pangolins are suggested to play such a role in SARS-CoV-2 transmission, with receptor binding domain of Pangolin-CoV being 91.02% identical to that of SARS-CoV-2 (5). However, this is an insufficient conclusion, as virus strains carried by civets and camels are over 99% identical in sequence to SARS-COV and MERS-CoV. To recapitulate, the knowledge of animal origin of SARS-CoV-2 is incomplete. The genome of the CoV family contains six open reading frames. The first ORF (ORF1a/b) encodes two non-structural polyproteins pp1a and pp1ab, the others being spike (S) glycoprotein, small envelope (E) protein, membrane (M) protein, and nucleocapsid (N) protein (6). People with COVID-19 manifest pneumonia with or without multi-organ diseases. The most common symptoms are fever, dry cough, and shortness of breath (7). The endpoint events vary, ranging from asymptomatic to multi-organ failure, even death (7). People of all groups are susceptible to COVID-19. According to a large observational study in China (8), 81% of patients had mild manifestations; 14% severe; 5% critical, which is defined as respiratory failure, septic shock, and multiple organ dysfunction.

## Physiological Changes During Pregnancy

Pregnant women undergo drastic changes both physiologically and immunologically. The uterus expands to meet the spatial demand of the ever-growing fetus. This is dominant in the late-mid and third trimesters, when the diaphragm is elevated and lungs are compressed, leaving compromised functional residual capacity, end-expiratory volumes, and residual volumes. Pregnant women also undergo significant changes in the coagulation system, where hypercoagulability prevents the failure of clotting as placenta flow is up to 700 ml min^−1^. As concluded from various reports, people with severe COVID-19 manifest coagulopathy, significantly elevated level of D-dimer (9) as well as decreased level of platelet count (9) and prolonged prothrombin time (10). Taken together, these findings coincide with what are considered physiological adaptations to pregnancy. Thus, it is believed that pregnant women are more susceptible to COVID-19 or are more likely to complicate the disease course of COVID-19. According to a large observational study based on the national surveillance system in the US (11), the admission rate of pregnant women was higher than that of the general population. Nevertheless, confounding factors, such as low admitting threshold from the perspective of healthcare givers, high chance of receiving tests and purpose of laboring, may account for this situation. Whether pregnancy is a risk factor for hospitalization or progression remains to be elucidated.

## Underlying Pathogenesis of SARS-CoV-2 Infection

SARS-CoV-2, like SARS-CoV, binds to the angiotensin-converting enzyme 2 (ACE2) receptor *via* S protein on its surface. ACE2 is widely distributed on the surface of nasal and bronchial epithelial cells and pneumocytes, making lung tissue an essential target to coronavirus. The affinity of S protein to ACE2 is 10 to 20 times that of SARS coronavirus, which partly explains why SARS-CoV-2 has stronger infectivity (12). The type 2 transmembrane serine protease (TMPRSS2), located near ACE2, is for S protein priming and aids viral entrance (13). After SARS-CoV-2, entrance through various pathways, the combination of pattern recognition receptor (PRR) to viral RNA signal synthesis of Interferon-*α* and *β*, which binds to its receptors on the uninfected cells in the vicinity to establish a cordon and leads to antiviral effects like degradation of both viral and host mRNA. This results in the overall reduction of protein synthesis, especially the expression of MHC proteins, making infected cells more susceptible to natural killer (NK) cells. However, recent studies have found that the infected epithelial cells upregulate ACE2, the entry for SARS-CoV-2, through interferon signals, which counteract the interferon’s antiviral effect at first (14). By recognizing “missing self” signals, NK cells kill virally infected cells and release IFN-*γ*, a vital cytokine responsible for enhancing phagocytosis of macrophages and antigen presentation of dendritic cells to T cells. Both of the latter secret cytokines responsible for the recruitment of T and B cells. The innate immune cells secrete pro-inflammatory cytokines and chemokines to augment inflammatory responses. Although the innate immune response plays an important role in initiating the antiviral response and the adaptive immune response, the adaptive immune system acts as the ultimate barrier that prevents virus replication and aids virus clearance (15). To be more specific, the steps mentioned above result in the formation of CD8^+^ T cells and CD4^+^ helper T cell, antigen-specific B cells. The th1-type immune response plays a leading role in the face of viral infection. The cytokine microenvironment produced by APC determines the direction of T cell responses. Helper T cells coordinate the overall adaptive response, and cytotoxic T cells are the key to killing virus-infected cells. The humoral immune response, especially the production of neutralizing antibodies, plays a protective role by limiting infection later to prevent future infections. Bost and colleagues (16) applied cRNA-seq and Viral-Track analysis to COVID-19 patient-derived samples to compare discrepant immune responses between mild and severe cases. They found that CD4^+^ helper T cell exhibited a relatively naïve phenotype in severe patients, and CD8^+^ T cells express a high profile of effector molecules in mild patients. Furthermore, genes of inflammatory cytokine and chemokines, genes associated with hypoxia and oxidative stress were upregulated, whereas MHC class II molecules and type I IFN genes were downregulated as it deteriorated.

Some clinically remarkable changes have been noticed between mild and severe cases, the first being neutrophil-to-lymphocyte ratio (NLR). High NLR is a marker predicting systemic inflammation and infection (17). Critical cases of COVID-19 were observed to exhibit a significant reduction in the quantity of peripheral CD8^+^ T cells with abnormal lymphopenia (18), which is consistent with what was found to be characteristic of severe sepsis and multiple organ failure (19). However, the underlying mechanism is still unclear. Higher expression levels of NKG2A on NK cells and CD8^+^ T cells were observed in severe cases of COVID-19 than that in mild cases, which means part of their functions were compromised in the face of viral infection; meanwhile, decreased secretion levels of IFN-*γ*, IL-2 and TNF-α were also noticed on NK cells and CD8^+^ T cells (20).

Cytokine release syndrome (CRS), also known as cytokine storm, manifests in the short term that the body secretes many cytokines, triggering systemic inflammatory response syndrome. High levels of pro-inflammatory cytokines may cause pulmonary edema, where there is infiltration of massive neutrophils, macrophages, and cytokines with alveolar damage. Clinical manifestations of CRS are high fever, hypotension, hypoxia and respiratory distress; organ dysfunction can also occur in severe cases, which requires intensive care and invasive ventilation. Studies have shown that compared with that of mild cases, plasma levels of IL2, IL6, IL7, IL10, G-SCF, IP10, MCP1, ferritin, and TNFα in severe cases of COVID-19 were elevated, indicating that the participation of these cytokines contributes to the pathogenesis of deterioration (21). Among them, IL-6 is mainly focused. IL-6 is produced by almost all stromal cells and immune cells. IL-6R, typically mIL-6R, is expressed on hepatocytes and immune cells; sIL-6R is secreted by activated T cells or cleaved from mIL-6R. The combination of IL-6 to IL-6R (whichever type) along with gp130 that transduces signals and is expressed on almost all cell types leads to activation of janus kinases-signal transducers and activators of transcription (JAK–STAT) pathway (22). JAKs are members of tyrosine kinases related receptors. It can transmit extracellular signals from upstream pro-inflammatory cytokines to activate STATs. The high affinity between extracellular signaling cytokines and their cognate receptors leads to receptor-related phosphorylation of JAK and STAT. Phosphorylated STATs form dimers, translocate into the nucleus, bind to DNA, and then transmit extracellular cytokine signals to transcription factor responses. IL-6 activates the JAK–STAT signaling pathway and confers various biological functions, including immune regulation, lymphocyte growth and differentiation, oxidative stress, *etc.* It is vital and somewhat protective in the acute and chronic phase of infection; however, the excessive level of IL-6 is associated with disease progression. Thus, IL-6 is considered a pleiotropic cytokine centered in CRS. Tocilizumab and sarilumab are IL-6 receptor antagonists that are used to treat rheumatoid arthritis. For COVID-19 patients who exhibit cytokine storms or secondary hemophagocytic lymphohistiocytosis (sHLH) accompanied by significantly increased levels of IL-6, ferritin, and D-dimer, these may be potential therapy (23). According to reports (24–27), the use of tocilizumab in patients with severe COVID-19 has reached favorable outcomes, including rapid but sustained improvement of clinical symptoms (28) in patients with acute respiratory failure, as well as decreased risk of non-invasive ventilation or mechanical ventilation (24). Tocilizumab can interfere with soluble and membrane-bound forms of IL-6 receptor, thereby blocking downstream activities. The sarilumab trial has just started in the United States, but data are yet to be published (29, 30). Therefore, IL-6 receptor antagonists are up-and-coming candidates in the treatment of severe COVID-19 patients. JAK inhibitors are also suggested as a promising approach (31).

Ferritin is another indicator elevated in severe cases. They are produced intracellularly and are secreted into serum within a physiological range of concentration. Ferritin loses most of its iron after secretion to serum. It is abnormally high in some inflammatory responses, which mainly results from the leakage of damaged cells. Circulating ferritin levels were found to be positively correlated with the degree of viral infection (32). It is noteworthy that ferritin *per se* is harmless, but the unliganded iron it loses is hydroxy radicals and can damage tissue. It is substantiated that during the acute phase of COVID-19 infection, massively produced pro-inflammatory cytokine including IL-6, TNF-α, and IL-12 stimulate hepatocytes and macrophages to secrete ferritin, which in turn contributes to the disease course (33). High ferritin levels are correlated with severe acute liver injury, intensive supportive care, and mechanical ventilation (34).

The immune status is shifted towards pro-inflammatory paradigm upon infection in healthy adults. On the contrary, pregnant women are deduced to be in immunosuppressive condition for the safety of semi-allograft fetuses, which increases the risk of being infected by the virus. However, such a classic host-graft model is challenged by some substantiation that maternal immunization of inactivated influenza vaccine can result in elevated antibody titers against influenza and predominant protection to infants (35). With the fact that mothers tolerate fetal-expressed paternal antigens, it is postulated that pregnancy gives an inert response to fetal-specific components, but acts at least usually, if not intensively, to other non-self ones, both locally and systemically (36, 37). The reasons for the immunological paradox of tolerance to a fetus with paternal antigens may incorporate many facets like a physical barrier of the placenta, decreased immunogenicity of fetal antigens, and unique immunological profile of maternal–fetal interface (37).

Since COVID-19 is a relatively new-onset pandemic that threatens millions of lives, its exact role on different trimesters of pregnancies is yet to be known. Here, we updated and summarized the effects of COVID-19 on pregnancy from the perspective of immunology.

## Immune Characteristics at Different Trimesters of Pregnancy

Pregnancy should be regarded as a developmental process with changing immunological profiles. Embryo implantation, placentation, fetal growth, parturition initiation, and so on, are associated with different immunological microenvironments. The first trimester is manifested as the pro-inflammatory process when the blastocyst recognizes the receptive endometrium and breaches the barrier. Fetus grows distinctly during the second trimester, favoring an anti-inflammatory profile, with Type 2 helper T cell (Th2) at its core. Labor initiation often comes after fetus maturation, which occurs in the third trimester. A switch back to pro-inflammatory status helps with this process.

The innate immunity in a pregnant woman is responsible for the maternal-to-fetal interface. Gaynor et al. (38) found that peripheral NK cells account for 5–30% of total circulating lymphocytes; decidual NK cells ≥70% in the first trimester. Both pNK and dNK decline with the advancement of gestation age. dNK is characteristic of low cytotoxicity because of their unique receptor expression profile that recognizes non-classical HLA on the extra-villous trophoblast and leads to subsequent immunotolerance. Macrophages are in immunomodulatory M2 phenotype throughout the maintenance of pregnancy and responsible for the phagocytotic activity of clearing apoptotic cells. They also secret proangiogenic factors like vascular endothelial growth factor (VEGF) and fibroblast growth factor (FGF) and remodeling factors such as MMP-3 and MMP-9 to help with extra-villous trophoblast invasion and spiral artery remodeling (39). DCs are downregulated in number and maturity with the advancement of normal pregnancy. Immature DCs are characterized by low secretion levels of pro-inflammatory cytokines like IFN-*γ* and IL-12, and low expression levels of classic HLA-DR phenotype, which are T cell co-activation signals. However, some studies disclosed that innate immune cells like NK, monocyte, and plasmacytoid dendritic cells elicit efficient cytokine responses in the face of the virus (40). TLRs 3, 7, 8, and 9 are mainly expressed at the syncytial trophoblast and amniotic layer at the maternal-to-fetal interface (41). T and B cells decline in frequency; naïve T cells’ ability to differentiate into mature T helper cells is compromised (42). All the evidences above pointed out that pregnancy is exquisite coordination of immune regulation.

## Pregnancy Outcomes at Different Trimesters

### First Trimester

Due to the limited time since the onset of the pandemic, there are still insufficient outcome data on neonates born to mothers infected by SARS-CoV-2 at the first trimester. However, it is proved that the stress-related atmosphere of the pandemic has increased recurrent pregnancy loss rates, though not significantly, indicating its potential psychological influence on fertility which requires special attention (43). Stefano Cosmaet al. (44) recruited 138 consecutive pregnant women attending first-trimester ultrasound screening and found cumulative incidence in the first trimester was about 10.1%. It is suggested that pregnancies at the first trimester are susceptible to COVID-19 with a high prevalence that is no different from other groups; a surveillance program should also be tailored to manage this cohort. However, generalization cannot be reached due to the limited sample size. The long-term adverse outcomes relating to early trimester infection and well-designed follow-up cohorts are needed for further evaluation and interpretation.

### Second Trimester

To date, data interpreting the causal relationship between mid-term SARS-CoV-2 infection and possible outcomes is lacking. It is found that neonates born to mothers with infection identified more than 14 days are less likely to yield positive nucleic acid tests (45).

### Third Trimester

Both COVID-NET (11) and SET-NET (45) found higher preterm birth rates in pregnancy with COVID-19 than that of the overall population, but causal linkage cannot be made due to not ruling out high iatrogenic rates. Except for preterm, vertical transmission is another issue that haunts obstetricians. Bioinformatics analysis (46) based on single-cell transcriptomic datasets of trophectoderm and placenta of all three trimesters revealed positive ACE2 and TMPRSS2 expression on both human trophectoderm and placenta, suggestive of possible intrauterine infection pathway. However, its interpretation should be taken with caution, for the presence of ACE2 and TMPRSS2 on the placenta does not necessarily mean an infection of the fetus. Though reports demonstrate a direct linkage between pregnancy with COVID-19 and neonatal infection, the rate is meager (47). Apart from intrauterine pathway, intrapartum infection, postpartum skin-to-skin touch, and breastfeeding are other ways that may result in neonatal infection. There is one case (48) demonstrating positive detection of SARS-CoV-2 in human breast milk. Still, conclusions cannot be made on the inability of mothers with COVID-19 to breastfeed. The threshold of SARS-CoV-2 mRNA quantification to infect a neonate is yet to be known; giving up breastfeeding does not always outweigh the risk of infection. Thus, a ban on breastfeeding is not recommended according to guidelines (49).

## Pregnant Women Are Excluded From Trials of Vaccination Against SARS-CoV-2

Before that, herd immunity is achieved by vaccines; facial masks, social distancing, and contact tracing are still main approaches to contain the pandemic. However, in some low-to-middle income areas, these are poorly managed and executed. As is mentioned above, pregnant women groups are at the same risks of getting infected. Trials testing the efficacy and safety of vaccines against SARS-CoV-2 exclude pregnant women groups (50). To recapitulate, though being at the same risk of exposure to SARS-CoV-2, pregnant women are less protected than other cohorts. Considerations regarding excluding pregnant women are as follows: 1) rising opportunity to gain vertical transmission pathways; 2) sparse data on following-up of neonates born to mothers infected with SARS-CoV-2 at different trimesters; 3) unforeseen adverse reactions related to vaccinations. An online survey of pregnant women and mothers of children under 18 conducted by Skjefte and colleagues (51) revealed that vaccine acceptance was geographically variated, being generally highest in India, the Philippines, and Latin America, but lowest in Russia, the United States, and Australia. Though knowing its importance, their primary concern is confidence in vaccine safety or effectiveness. Thus, countries should get underway to collaborate on data regarding concerning issues of this unique cohort. Until now, there is one study recruiting healthy pregnant women that are willing to get vaccinated against COVID-19 (52), which is about to yield data of vital preciousness and importance.

## Discussion

Pregnancy is an exquisite coordination of immune response at different stages. Instead of presuming it to be an immunosuppressive status, accumulating evidence suggests that pregnant women have a robust immune response to non-fetal-specific antigens. Obstetric outcomes of pregnancy with COVID-19 include spontaneous and iatrogenic preterm birth, vertical transmission with low probability, pregnancy loss, and increased cesarean section rate (11, 45). Though being at the same risk of infection, pregnant women are excluded cohorts from participating in the trial of vaccination against SARS-CoV-2, due to ethical and lack of evidence. Thus, future follow-up studies focusing on maternal and neonatal outcomes are desperately needed to provide evidence for including pregnant women.

## Author Contributions

DD, YZ, and SL contributed to the study design. DL, JP, RL, XL, RW, DW, and MH collected and analyzed the data. DL and RW interpreted the results. DL, DD, and SL wrote the manuscript. All authors contributed to the article and approved the submitted version.

## Funding

This study was supported by the National Science and Technology Pillar Program of China during the Twelfth Five-Year Plan Period (grant No.2014BAI05B05), the National Clinical Research Center for Obstetrics and Gynecology (2015BAI13B05); the National Natural Science Foundation of China (81672085; 81372804; 81873843); the Chinese Medical Association of Clinical Medicine special funds for scientific research projects (17020400709); the Fundamental Research Funds for the Central Universities (grant No.2017KFYXJJ102 and 2019KFYXKJC053)the Hubei Provincial Natural Science Foundation of China (2019CFA062); the Strategic Collaborative Research Program of the Ferring Institute of Reproductive Medicine, Ferring Pharmaceuticals and Chinese Academy of Sciences (FIRMSCOV05). The authors declare that this study received funding from Ferring Pharmaceuticals. The funder was not involved in the study design, collection, analysis, interpretation of data, the writing of this article or the decision to submit it for publication.

## Conflict of Interest

The authors declare that the research was conducted in the absence of any commercial or financial relationships that could be construed as a potential conflict of interest.
